# Computational Insights into the Binding Mechanism of OxyS sRNA with Chaperone Protein Hfq

**DOI:** 10.3390/biom11111653

**Published:** 2021-11-08

**Authors:** Mengxin Li, Yalong Cong, Yifei Qi, John Z. H. Zhang

**Affiliations:** 1 Shanghai Engineering Research Center of Molecular Therapeutics and New Drug Development, Shanghai Key Laboratory of Green Chemistry & Chemical Process, School of Chemistry and Molecular Engineering, East China Normal University at Shanghai, Shanghai 200062, China; 1716504544@qq.com (M.L.); yalongcong@163.com (Y.C.); yfqi@chem.ecnu.edu.cn (Y.Q.); 2 Shenzhen Institute of Synthetic Biology, Shenzhen Institutes of Advanced Technology, Chinese Academy of Sciences, Shenzhen 518000, China; 3 NYU-ECNU Center for Computational Chemistry at NYU Shanghai, Shanghai 200062, China; 4 Department of Chemistry, New York University, New York, NY 10003, USA

**Keywords:** small RNA OxyS, RNA chaperone Hfq protein, gene expression regulator, molecular dynamics simulations, binding free energy, interaction entropy

## Abstract

Under the oxidative stress condition, the small RNA (sRNA) OxyS that acts as essential post-transcriptional regulators of gene expression is produced and plays a regulatory function with the assistance of the RNA chaperone Hfq protein. Interestingly, experimental studies found that the N48A mutation of Hfq protein could enhance the binding affinity with OxyS while resulting in the defection of gene regulation. However, how the Hfq protein interacts with sRNA OxyS and the origin of the stronger affinity of N48A mutation are both unclear. In this paper, molecular dynamics (MD) simulations were performed on the complex structure of Hfq and OxyS to explore their binding mechanism. The molecular mechanics generalized born surface area (MM/GBSA) and interaction entropy (IE) method were combined to calculate the binding free energy between Hfq and OxyS sRNA, and the computational result was correlated with the experimental result. Per-residue decomposition of the binding free energy revealed that the enhanced binding ability of the N48A mutation mainly came from the increased van der Waals interactions (vdW). This research explored the binding mechanism between Oxys and chaperone protein Hfq and revealed the origin of the strong binding affinity of N48A mutation. The results provided important insights into the mechanism of gene expression regulation affected by protein mutations.

## 1. Introduction

In bacteria, the small RNA (sRNA) which cannot be encoded into protein owing to the absence of open reading frames plays a critical role in the regulation of gene expression [1,2,3,4]. Under environmental stresses such as oxidative stress, low temperature, iron ion concentration, and so on, different types of sRNA are produced to regulate the expression of genes by targeting different messenger RNA (mRNA) and affecting their structures as well as translation efficiency [5,6,7]. In this process, the target mRNA can encode proteins in response to external changes and hostile environments. Based on the gene location, sRNA can be divided into two classes [8]: cis-encoded sRNA and trans-encoded sRNA. The cis-encoded sRNA is transcribed from the DNA strand complemented with target mRNA, promoting translation [9,10]. The trans-encoded regulatory sRNA is transcribed from another genomic region, regulating the stability of the target mRNA by antisense mediation mechanism [11]. The latter, referred to as trans-encoded sRNA orchestrate dynamics response, most strongly depend on the RNA chaperone Hfq that facilitates the formation of base pair between sRNA and target mRNA [12,13,14], and may change the stability and structure of the interacted RNA.

The Hfq protein is a ring-shaped RNA-binding protein of the Sm/LSm family that consists of six identical subunits [15,16,17,18], in which each subunit includes a β5-α1-β-β2-β3-β4 conformation. The N-terminal structural motifs of the six subunits form a ring-shaped core region. Structural studies have revealed that Hfq protein has three different binding sites: the proximal face, the distal face, and the lateral rim [19,20]. The proximal binding site, located on one side of the ring, prefers to bind U-rich single-stranded RNA (ssRNA) while the distal binding site prefers A-rich ssRNA. The lateral rim can bind to A-rich and U-rich RNA substrates. The mutation of the Hfq gene can reduce the adaption of bacteria in a dynamic environment and cause defects in growth rate and tolerance of stress [21,22,23,24,25]. The ability of Hfq to influence the living process for bacteria is likely attributed to the role of the sRNA chaperone protein. In vivo, the mutation of Hfq can affect the accumulation of the interacted sRNA, resulting in the change of the translation efficiency for mRNA, which in turn affects the expression of the gene.

Under the oxidative stress condition, the expression of OxyS sRNA is increased. As a regulator of pleiotropy, OxyS can regulate the gene expression by up-regulating and down-regulating a variety of genes with the help of chaperone protein Hfq. A-rich sequence between stem-loops b and c of OxyS sRNA was discovered as the binding region with Hfq protein [26]. To understand the mechanism of how sRNA regulates mRNA, it is important to understand how Hfq binds to sRNA. However, the interaction mechanism between Hfq and sRNA at atomic levels is unclear. Wartell et al. [27] studied the interaction of Hfq with sRNA mRNAs, and concluded that the mechanism of the antisense regulation of OxyS for mRNA was not by the binding to rpoS mRNA but by sequestering Hfq. Callaghan et al. [28] discovered that the Hfq protein did not change its own conformations obviously in the process of interacting with RNA and resulted in the alternation of sRNA structure, showing the importance of exploring the structure change of sRNA induced by Hfq protein. Barabas et al. [14] presented two Hfq-A18 complexes and showed that base stacking can facilitate the interaction of target mRNA with Hfq-bound sRNA with (ARN)X motifs, putting forward to the interaction details. Shi et al. found that the N48A mutation of Hfq protein did not weaken the interaction of Hfq and ssRNA while dramatically enhance the binding ability, causing sRNA–mRNA regulatory defects in the experiment [29]. This phenomenon raises the question of whether N48 plays an important role in balancing Hfq-OxyS and how the binding capacity of Hfq and OxyS influences the regulation function of the gene. However, only a few researchers explore the detailed interaction of Hfq and OxyS linker region at an atomic level to understand the regulatory mechanism, especially with molecular dynamics (MD) simulations.

Compared with experiments, MD [30,31] simulations can provide more detailed dynamic information and have been widely used in the study of the structure and function of biomolecules. For biological macromolecule systems, binding free energy is crucial to study binding mode and interaction strength. In binding free energy calculations, the accurate calculation of entropic contribution is difficult [32]. Recently, an interaction entropy (IE) method [33,34,35] was proposed for the efficient calculation of entropy in protein-ligand and protein-protein bindings [33,34,35,36]. In the present study, the Molecular Mechanics/Generalized Born Surface Area (MM/GBSA) and IE methods were combined to explore detailed binding mode and gene expression regulation mechanism of two A-rich fragments 5′-AACUAAA-3′ and 5′-AUAACUA-3′ of the OxyS linker region with the Hfq protein (hereafter referred to as Ads system and Aus system). Moreover, in order to obtain a more accurate and rigorous assessment of the impact of the N48A mutation, the free energy perturbation (FEP) method [37] was used to calculate the relative binding free energy.

## 2. Materials and Methods

### 2.1. Molecular Dynamics Simulations

The initial crystal structures for the Ads (PDBID: 4QVD) and Aus systems (PDBID: 4QVC) [29] were obtained from the Protein Data Bank (PDB) [38]. The structure of the N48A mutant was obtained by performing mutation in the Leap module of AMBER18. The missing nucleotides were filled by PDBfixer. Please see https://github.com/openmm/pdbfixer (accessed on 10 May 2020). The AMBER ff14SB and YIL force fields were used to parameterize the protein and RNA [39]. Then the complex was solvated in a TIP3P [40] water box with a buffer distance of 12 Å. The counter ions were added to keep the system neutral. In order to relax the system, two steps of energy minimization were performed. First, the system was minimized by 5000 steps steepest descent followed by 5000 steps conjugate gradient minimization. In this process, solute was restrained with a force constant of 500 $\mathrm{kcal}/\left(\mathrm{mol}\cdot {Å}^{2}\right)$. Second, the whole system was relaxed with a weak 1$\;\mathrm{kcal}/\left(\mathrm{mol}\cdot {Å}^{2}\right)$ harmonic constraints on backbone atoms of RNA. Then the whole system was heated from 0 to 300 K in 300 ps with the 5 $\mathrm{kcal}/\left(\mathrm{mol}\cdot {Å}^{2}\right)$ harmonic constraint on solute. Finally, two-stage MD simulations in the NPT ensemble were carried out for 100 ns in total. The first 50 ns simulation was performed for system equilibration and the second 50 ns was for production runs. During the simulation process, 1 $\mathrm{kcal}/\left(\mathrm{mol}\cdot {Å}^{2}\right)$ harmonic constraints were included in the backbone of RNA. The trajectories were saved every 1 ps. The long-range electrostatic interactions were calculated by the particle mesh Ewald (PME) [41] and SHAKE [42] algorithm was adopted to deal with all bonds involving hydrogen atoms. A cut-off distance of 10 Å was used to deal with the non-bonded interactions. The Langevin thermostat and Berendsen barostat were used to control the temperature and pressure.

### 2.2. MM/GBSA Method

The MM/GBSA approach was used to calculate the enthalpy of the binding free energy. The formula could be expressed by the following terms:

(1) $$\Delta G_{bind}=G_{complex}-G_{protein}-G_{RNA}$$

where the $G_{complex}$, $G_{protein}$ and $G_{RNA}$ represented the free energy of complex, protein and RNA, respectively. The binding free energy also could be calculated by the following equation:

(2) $$\Delta G_{bind}=\Delta H-T\Delta S$$

In which ΔH and −TΔS represented enthalpy change and entropy change, respectively. Among them:

(3) $$\Delta H=\Delta E_{vdw}+\Delta E_{ele}+\Delta G_{gb}+\Delta G_{np}$$

where $\Delta E_{vdw}$ and $\Delta E_{ele}$ represented van der Waals (vdW) interaction and electrostatic interaction between the protein and the RNA. $\Delta G_{gb}$ and $\Delta G_{np}$ represented polar solvation free energy and non-polar solvation free energy, respectively.

The $\Delta G_{gb}$ term was calculated by the Generalized Born (GB) model, and the non-polar solvation free energy could be obtained by the empirical solvent accessible surface (SASA) formula:

(4) $$\Delta G_{np}=\gamma SASA+\beta$$

The values of γ and β were 0.005 $\mathrm{kcal}/\left(\mathrm{mol}{Å}^{2}\right)$ and 0.000 kcal/mol.

### 2.3. Interaction Entropy

The IE method [43] was used to calculate the entropy contribution and the formula was given by

(5) $$-T\Delta S=KTln\langle e^{\beta \Delta E_{int}}\rangle$$

where $\Delta E_{int}$ represented the fluctuation of the gas-phase interaction energy:

(6) $$\Delta E_{int}=E_{int}-\langle E_{int}\rangle$$

In the process of numerical integration, to eliminate the energy “noises”, energy points that were three standard deviations away from the average were discarded. Finally, the binding free energy was obtained by the summation of enthalpy calculated by MM/GBSA and the entropy was calculated by the IE method.

### 2.4. Free Energy Perturbation Calculations

To more accurately compute the relative binding free energy between the wild type and the mutant species, the free energy perturbation (FEP) was employed in the calculation. In the FEP method, the relative binding free energy ΔΔG between the mutant and the wild type was given by

(7) $$\Delta \Delta G=\underset{\lambda }{\sum }\Delta G_{\lambda }$$

where $\Delta G_{\lambda }$ was computed as

(8) $$\Delta G_{\lambda }=-KTln\langle e^{-\frac{V_{\lambda +\Delta \lambda }-V_{\lambda }}{KT}}\rangle {}_{\lambda }$$

where K and T were Boltzmann constant and absolute temperature. $V_{\lambda }$ was defined as $V_{\lambda }=\left(1-\lambda \right)V_{wt}+\lambda V_{mut}$ and λ was the coupling parameter that varied from 0 and 1.

The mutated residue was treated as a softcore region and a total of 11 λ-windows were used. In each λ-window, the initial structure was optimized by 25,000 steps of steepest descent followed by 25,000 steps of conjugate gradient minimization. Then the system was heated from 0 K to 300 K. Then, a 3-ns MD was performed and 2000 snapshots were used for energy calculation.

## 3. Results and Discussion

### 3.1. MD Simulation and Binding Free Energies

To reduce the statistical errors, three independent 100-ns trajectories with random initial velocities were generated for each complex system. The root means square deviation (RMSD) of the backbone atoms in Hfq and OxyS sRNA relative to the initial structure for all systems were calculated to ensure that the systems had reached equilibrium during the simulation (Figures S1–S4). In the 100-ns simulation, the first 50 ns was used for the convergence of the structure, and the last 50 ns was used for analysis. In order to obtain better convergence of the IE method, the root means square fluctuation (RMSF) of the gas-phase interaction energy was calculated as a function of time (Figures S5 and S6) to measure the fluctuation of next 10-ns samples, as following:

(9) $$RMSF(t)=\sqrt{{\langle (E_{int}-\langle E_{int}\rangle )}^{2}\rangle }$$

where $\langle E_{int}\rangle$ represents average gas-phase interaction energy of next 10-ns samples.

Based on the RMSF calculation, the 10-ns window with the smallest fluctuation was used for further energy calculation and analysis. Besides, the entropy of a 10-ns window with the smallest gas-phase energy fluctuation for all systems was also depicted in Figures S7 and S8, showing that the entropy changes over time had a good convergence.

The detailed energies of each trajectory were summarized in Table 1. The binding free energy of three simulations was basically consistent within the acceptable error range. The average binding free energy of Ads, Ads (N48A), Aus, and Aus (N48A) were −27.7, −29.9, −29.7, and −31.3 kcal/mol, which were in line with the trend of experimental values of −8.0, −9.6, −10.5, and −10.6 kcal/mol. The calculated affinity of Aus was stronger than that of Ads, and the affinities of Ads (N48A) and Aus (N48A) mutants were also stronger than the respective wild types, which were in agreement with the experimental trend and demonstrated that the simulation and calculated method were reliable.

### 3.2. Comparison of Binding Mode of Ads and Aus

**Figure 1:**
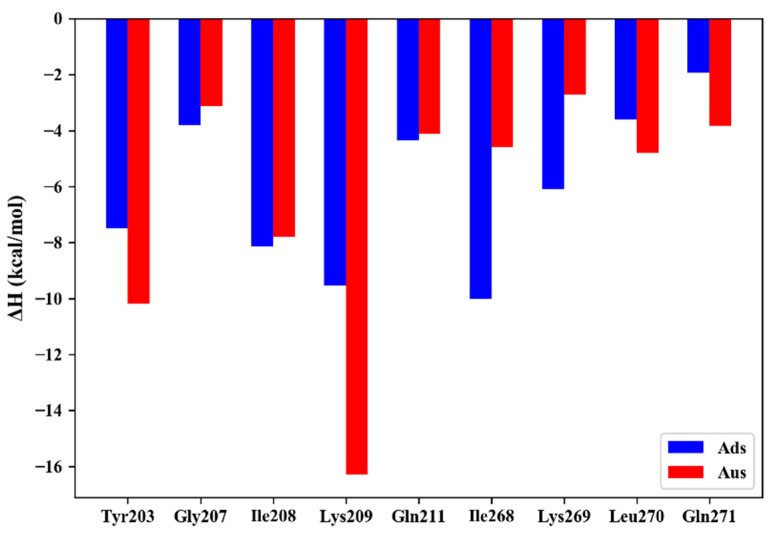
The key residues that contribute more than −2.5 kcal/mol in Ads and Aus.

Experimental studies found that the binding affinities of Ads and Aus differ by 2.5 kcal/mol despite the high similarity of the RNA sequences. In order to explore the reason behind the stronger binding ability in the Aus system, residue decomposition was performed to measure the per-residue contribution. The key residues in Ads and Aus that contributed more than −2.5 kcal/mol were plotted in Figure 1. The stronger binding affinity in the Aus system mainly came from residues Tyr203 (−10.2 kcal/mol in Aus vs. −7.5 kcal/mol in Ads) and Lys209 (−16.3 kcal/mol vs. −9.5 kcal/mol). It was worth noting that residues Ile268 and Lys269 had a stronger binding affinity in Ads compared with the Aus system.

**Figure 2:**
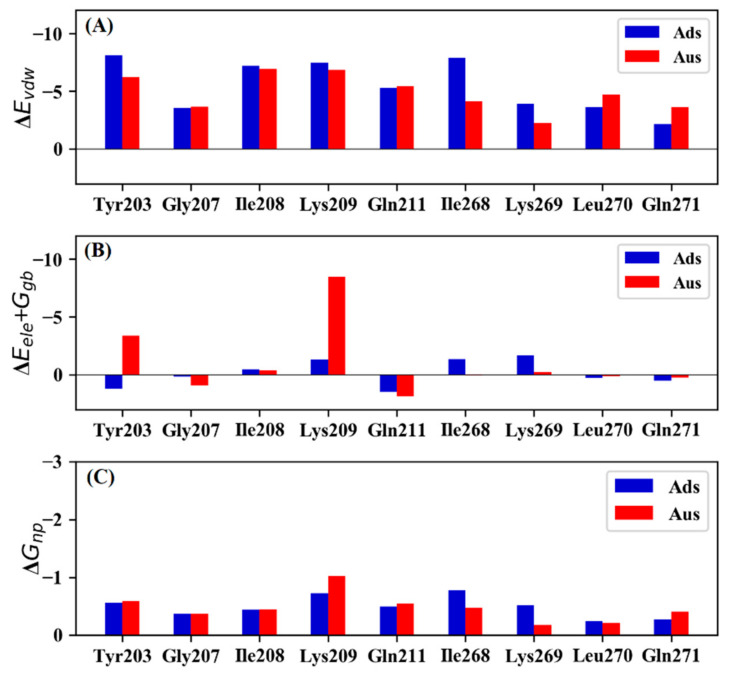
Detailed energy decomposition of Ads and Aus. (**A**) van der Waals (vdW). (**B**) sum of electrostatic and polar solvation energy. (**C**) non-polar solvation energy.

To further understand the origin of the binding energy difference, the contribution of key residues in the Ads and Aus systems were decomposed into vdW, electrostatic, polar solvation energy, and non-polar solvation energy (Figure 2). Compared with Ads, the favorable contribution for Tyr203 and Lys209 in Aus mainly came from the electrostatic interaction and polar solvation energy. The main source for the weaker interaction of Ile268 in Aus mainly originated from the vdW. For Lys269, the weaker energy mainly came from vdW and electrostatic interaction.

**Figure 3:**
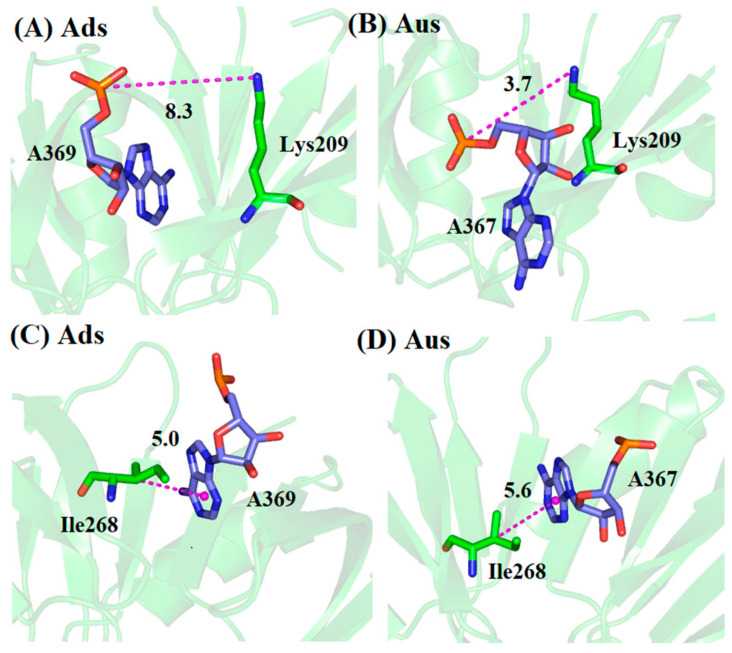
The conformations in the lowest-energy structures of MD trajectory for key residues Lys209 and Ile268. (**A**) Lys209 in Ads system. (**B**) Lys209 in Aus system. (**C**) Ile268 in Ads system. (**D**) Ile268 in Aus system.

**Figure 4:**
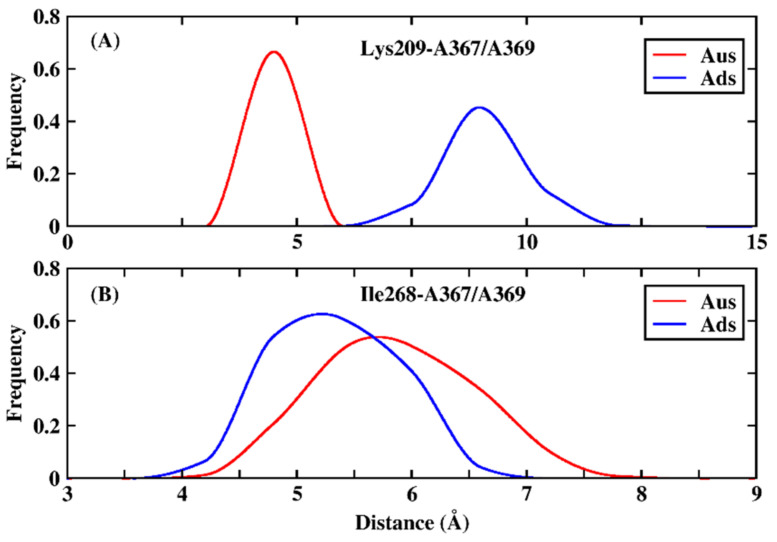
The distribution of distance for key residue Lys209 and Ile268 in Ads and Aus system. (**A**) the distance between the charged group of Lys209 and the phosphate group of adenine nucleotide A367 of Aus and A369 of Ads system. (**B**) distance between the alkyl of residue Ile268 and the hydrophobic ring of adenine nucleotide A367 of Aus and A369 of Ads system.

**Figure 5:**
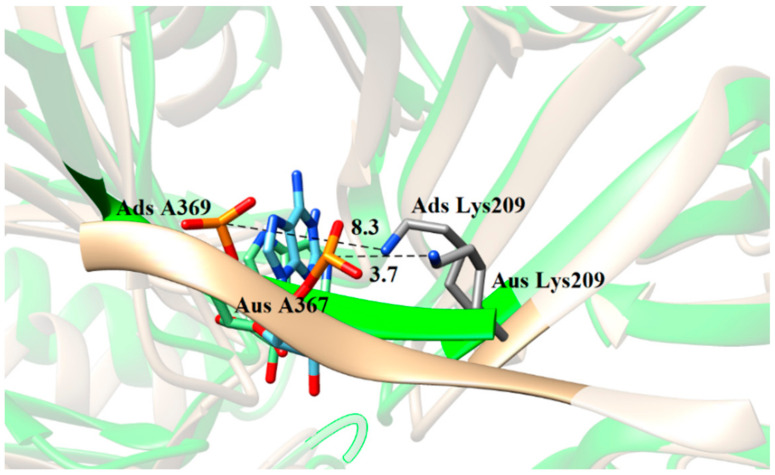
The superposition of Ads and Aus structure in the lowest-energy structures of MD trajectory (Aus in gold, Ads in green). The distance (Å) between the charged group of Lys209 and the phosphate group of adenine nucleotide A367 in Aus system and A369 in Ads system was labeled.

To further explore the origin of the favorable and weak residues, a hydrogen bond analysis based on MD trajectory was performed (Table 2). The favorable electrostatic interaction for Tyr203 in Aus mainly arose from the hydrogen bond between Tyr203 and adenine nucleotide A367, with the occupancy of 95.5%, whereas the same hydrogen bond did not form in Ads. Similarly, residue Lys269 formed a hydrogen bond in Ads with the occupancy of 62.1% but did not form any hydrogen bond in Aus, resulting in the stronger electrostatic interaction in Ads. Besides this, the average hydrogen bond length between the backbone N atom of Lys269 and OP1 atom of uracil nucleotide U366 in Aus was unusually large (11.9 Å), which also resulted in weaker vdW interaction. For residues Lys209 and Ile268, the simulated configurations were analyzed in Figure 3. The charged group of Lys209 formed a salt bridge with the phosphate group of sRNA in Aus with an average distance of 3.7 Å while the corresponding average distance formed in Ads was 8.3 Å (Figure 3A,B). The frequency distribution of the distance between the charged group of Lys209 and phosphate group of sRNA also showed this obvious phenomenon (Figure 4A). In order to explore the origin of the difference in structure, the superimposition of the conformation based on the lowest energy structure was depicted (Figure 5). The phosphate group of nucleotides moved away from Lys209 in the Ads system relative to the Aus system, making it impossible to form the same salt bridge in the Ads system. As for Ile268, the vdW hydrophobic interaction in Ads was significantly stronger than that of Aus. According to the structural analysis in Figure 3C,D, the alkyl of Ile268 formed CH-π interaction with A369 of Ads and A367 of Aus, in which the distance between alkyl with the center of mass of nucleobases was 5.0 and 5.6 Å in Ads and Aus, respectively. The shortened distance in Ads was the reason for the higher vdW interaction. Besides this, the distribution of the distance in Figure 4B also showed a shortened distance in Ads. Although Ile268 and Lys269 had stronger enthalpy in Ads, the increased values did not exceed the stronger contribution of residues Tyr203 and Lys209 in Aus.

### 3.3. Mutational Effect of Ads and Aus

Experimental studies showed that the N48A mutation improved the binding affinity for both Ads and Aus [29]. According to the calculated values based on the combination of MM/GBSA and IE (Table 1), the average binding free energy of Ads (N48A) and Aus (N48A) were stronger 2.2 and 1.6 kcal/mol compared with that of the wild type. In order to further verify the reliability of our results, the relative binding free energy between wild and mutated systems was calculated using FEP methods (Table 3). The mutation enhanced the binding in Ads and Aus, with the values of 2.9 and 4.1 kcal/mol, which was in line with the experiment trends and basically consistent with the results calculated by MM/GBSA and IE, indicating that our results were reliable. Because the FEP method simulated a series of nonphysical intermediate states and could not provide detailed energy information to deeply explore the interaction mechanism, the following analyses of detailed atomic interactions were based on the results calculated by MM/GBSA.

**Figure 6:**
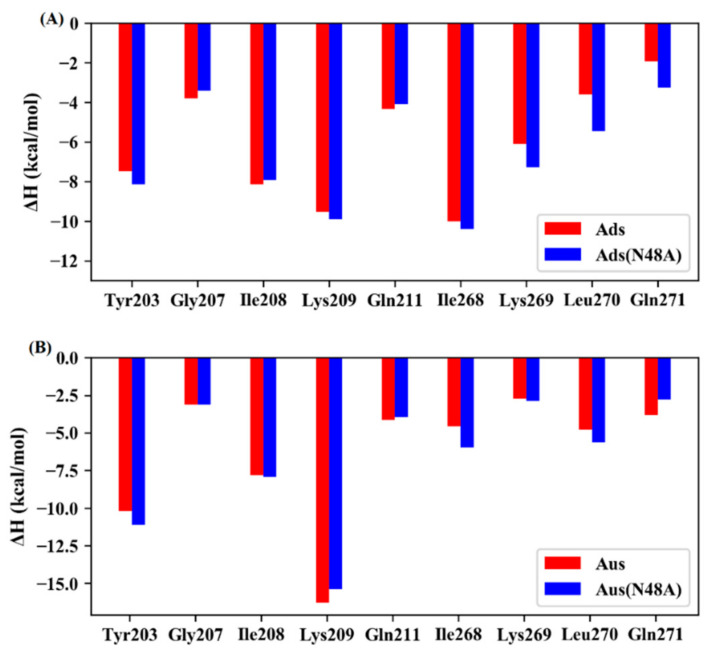
Key residues in the wild type and mutated systems. (**A**) Ads and Ads (N48A). (**B**) Aus and Aus (N48A).

In order to obtain more detailed interaction information and reveal the molecular mechanism of the mutational effect, the residue-based free energy decomposition method was applied to the wild-type and mutant systems (Figure 6). The stronger binding affinity in Ads (N48A) mainly came from the residues Lys269, Leu270, and Gln271. For Aus (N48A), Tyr203, Ile268, and Leu270 were the main beneficial residues for the stronger binding ability. It was worth noting that Gln271 contributed more than 1 kcal/mol in Aus than that in Aus (N48A).

**Figure 7:**
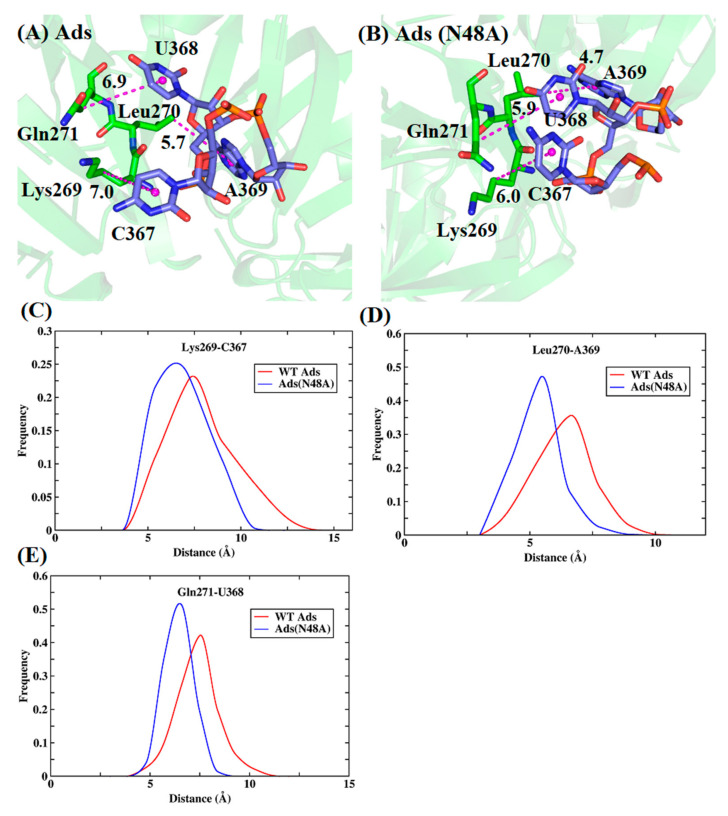
The conformations of the lowest-energy structures and distance distribution of key residues in the Ads and Ads (N48A). (**A**) Ads. (**B**) Ads (N48A). (**C**–**E**) distance distribution between alkyl of Lys269 and hydrophobic ring of C367 (**C**), alkyl of Leu270 and hydrophobic ring of A369 (**D**), and alkyl of Gln271 and hydrophobic ring of U368 (**E**).

**Figure 8:**
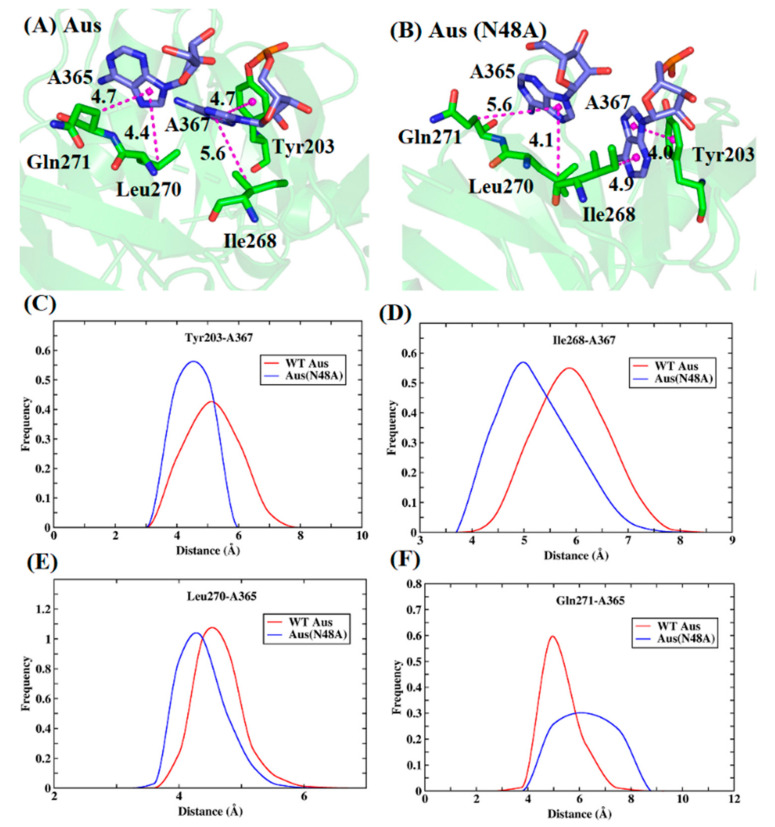
The conformations of the lowest-energy structures and distance distribution of key residues in the Aus and Aus (N48A). (**A**) Aus. (**B**) Aus (N48A). (**C**–**F**) distance distribution between benzene ring of Tyr203 and hydrophobic ring of A367 (**C**), alkyl of Ile268 and hydrophobic ring of A367 (**D**), alkyl of Leu270 and hydrophobic ring of A365 (**E**), and alkyl of Gln271 and hydrophobic ring of A365 (**F**).

To further explore the mechanism of the affinity change and obtain a more intuitive comparison, the conformations of these residues were plotted in Figure 7 and Figure 8 based on the lowest-energy structures from MD simulation. The interaction of Lys269 with RNA mainly came from the alkyl of the residue and the hydrophobic ring of C367, with the contribution of −6.0 and −7.2 kcal/mol in Ads and Ads (N48A) (Figure 6A and Figure 7). From the energy decomposition analyses, the increased energy mainly depended on the vdW interaction. The average distance of alkyl with the hydrophobic ring was 7.0 Å in Ads while the value was 6.0 Å in Ads (N48A). Similarly, the interaction of residue Leu270 with RNA mainly came from the interaction of alkyl and the hydrophobic ring of A369, which provided an average contribution of −3.6 and −5.4 kcal/mol in Ads and Ads (N48A). The average distance of alkyl and the center of mass of the base was 5.7 Å in Ads and 4.7 Å in Ads (N48A), causing the increase in the vdW interaction. The interaction of residue Gln271 with RNA was mostly contributed by CH-π interaction. After the mutation, the enthalpy changed from −1.9 to −3.2 kcal/mol. The increased energy was mainly attributed to the vdW interaction in which the distance of alkyl with the hydrophobic ring of U368 was shortened from 6.9 to 5.9 Å. Moreover, the peaks of distance distribution for Lys269, Leu270, and Gln271 with the interacted nucleotide were all smaller in Ads (N48A) compared with Ads (Figure 7C–E).

The binding modes between key residues and RNA in Aus were similar to those in Ads. The benzene ring of Tyr203 formed π- π hydrophobic interaction with the hydrophobic ring of A367 (Figure 8A,B). After the N48A mutation, as the distance of the benzene ring with the base was shortened from 4.7 to 4.0 Å, the enthalpy increases from −10.1 to −11.1 kcal/mol. Besides this, the alkyl of Ile268 and Leu270 formed CH-π hydrophobic interaction with the nitrogenous base of RNA. The contribution of Ile268 and Leu270 in Aus was −4.5 and −4.7 kcal/mol while the contribution in Aus (N48A) changed to −5.9 and −5.6 kcal/mol, with the increased energy −1.4 and −0.9 kcal/mol. The average distance between the center of mass of nitrogenous base and alkyl of Ile268 was 5.6 and 4.9 Å in Aus and Aus (N48A). For Leu270, the distance was 4.4 and 4.1 Å, respectively. The shortened distance in Aus (N48A) led to stronger vdW interaction. There was a noticeable residue Gln271 with lower energy in mutant compared with wild type. Because of the conformational change caused by the mutation, the distance of the alkyl group with nitrogenous base was increased from 4.7 to 5.6 Å, causing weaker vdW interactions. From the distance distribution in Figure 8C–F, the distance peaks for above mentioned stronger residues with their own interacted nucleotide in Aus (N48A) were all smaller than Aus. The above analyses suggested that the N48A mutation can affect the conformations of the surrounding residues, which led to a stronger interaction with sRNA.

## 4. Conclusions

In this paper, MD simulation and binding free energy calculation were performed to explore the binding mechanism of sRNA (OxyS) and chaperone protein (Hfq). The calculated binding free energy from MM/GBSA and IE was consistent with experimental results. The decomposition of per-residue energy showed that Tyr203 and Lys209 played key roles in the favorable interaction of Aus compared with Ads, in which the electrostatic interaction was the major driving force. Moreover, the stronger binding affinity of the N48A mutants relative to the wild types in both Ads and Aus mainly came from vdW hydrophobic interactions. Structural analyses indicated that the mutation changed the conformation of surrounding residues.

This study provided an atomic-level interaction mechanism of sRNA and Hfq based on dynamics information, and it deepened our understanding of the binding affinity change caused by the mutation. It was expected that the results could provide useful information for modifying the protein by analyzing the interaction between the Hfq protein and RNA.

## Figures and Tables

**Table 1:** The experimental and calculated binding free energy from three MD trajectories. The experimental values are from Shi et al. [29]. All energy values are given in kcal/mol).

System	Simulation	ΔH	−TΔS	$\Delta G_{cal}$	$\Delta G_{exp}$
Ads	No. 1	−62.4 ± 0.4	33.8 ± 0.0	−28.6 ± 0.4	−**8.0**
	No. 2	−60.3 ± 0.5	35.1 ± 0.0	−25.2 ± 0.5	
	No. 3	−62.4 ± 0.5	33.3 ± 0.0	−29.2 ± 0.5	
	Ave	−61.7	34.0	**−27.7**	
Ads (N48A)	No. 1	−65.4 ± 0.4	36.5 ± 0.0	−28.9 ± 0.4	−**9.6**
	No. 2	−66.5 ± 0.4	38.2 ± 0.0	−28.3 ± 0.4	
	No. 3	−64.1 ± 0.3	31.5 ± 0.0	−32.6 ± 0.3	
	Ave	−65.3	35.4	**−29.9**	
Aus	No. 1	−61.3 ± 0.4	30.7 ± 0.0	−30.6 ± 0.4	−**10.5**
	No. 2	−64.2 ± 0.4	32.7 ± 0.0	−31.5 ± 0.4	
	No. 3	−67.5 ± 0.4	40.4 ± 0.0	−27.2 ± 0.4	
	Ave	−64.3	34.6	**−29.7**	
Aus (N48A)	No. 1	−68.7 ± 0.4	37.8 ± 0.0	−30.9 ± 0.4	−**10.6**
	No. 2	−67.7 ± 0.3	33.3 ± 0.0	−34.4 ± 0.3	
	No. 3	−65.8 ± 0.4	37.1 ± 0.0	−28.7 ± 0.4	
	Ave	−67.4	36.1	**−31.3**	

Bold values emphasize the numbers that are compared with experiment.

**Table 2:** Occupancy of hydrogen bonds for Ads and Aus.

	Acceptor	Donor	Occupancy (%)	Distance (Å)	Angle (°)
Ads	A369@OP2 ^a^	Tyr203@OH	0.0	5.3	111.0
	U368@OP1	Lys269@N	62.1	3.8	154.8
Aus	A367@OP2 ^a^	Tyr203@OH	95.5	2.7	163.4
	U366@OP1	Lys269@N	0.0	11.9	132.9

^a^ A367 in Aus corresponds to A369 in Ads.

**Table 3:** The differences in binding free energy caused by the N48A mutation calculated with MM/GBSA/IE and FEP. All values are in kcal/mol.

System	$\Delta \Delta G_{MM/GBSA/IE}$	$\Delta \Delta G_{FEP}$	$\Delta \Delta G_{exp}$
Ads (N48A)	−2.2	−2.9	−1.6
Aus (N48A)	−1.6	−4.1	−0.2

## Supplementary Materials

The following are available online at https://www.mdpi.com/article/10.3390/biom11111653/s1, Figure S1. Protein and RNA RMSD of the Ads system from three simulations., Figure S2. Protein and RNA RMSD of the Ads(N48A) system from three simulations, Figure S3. Protein and RNA RMSD of the Aus system from three simulations, Figure S4. Protein and RNA RMSD of the Aus(N48A) system from three simulations, Figure S5. The RMSF of gas phase interaction energy of 10-ns sliding window in the last 50 ns for Ads and Ads(N48A) systems. The x-axis represents the start time of a 10-ns window. The windows with the smallest RMSF are marked with triangles, Figure S6. The RMSF of gas phase interaction energy of 10-ns sliding window in the last 50 ns for Aus and Aus(N48A) systems. The x-axis represents the start time of a 10-ns window. The windows with the smallest RMSF are marked with triangles, Figure S7. Time-averaged interaction entropy of continuous 10 ns with lowest energy fluctuation of for Ads and Ads(N48A) systems under three simulations, Figure S8. Time-averaged interaction entropy of continuous 10 ns with lowest energy fluctuation of for Aus and Aus(N48A) systems under three simulations.
