# Supplementary material for: Computational Insights into the Binding Mechanism of OxyS sRNA with Chaperone Protein Hfq
Source: Biomolecules. 2021 Nov 8;11(11):1653. doi: 10.3390/biom11111653 (PMC8615722; doi:10.3390/biom11111653)
Supplement: Supplementary file 1 [file biomolecules-11-01653-s001.zip › biomolecules-1407410-supplementary.pdf]

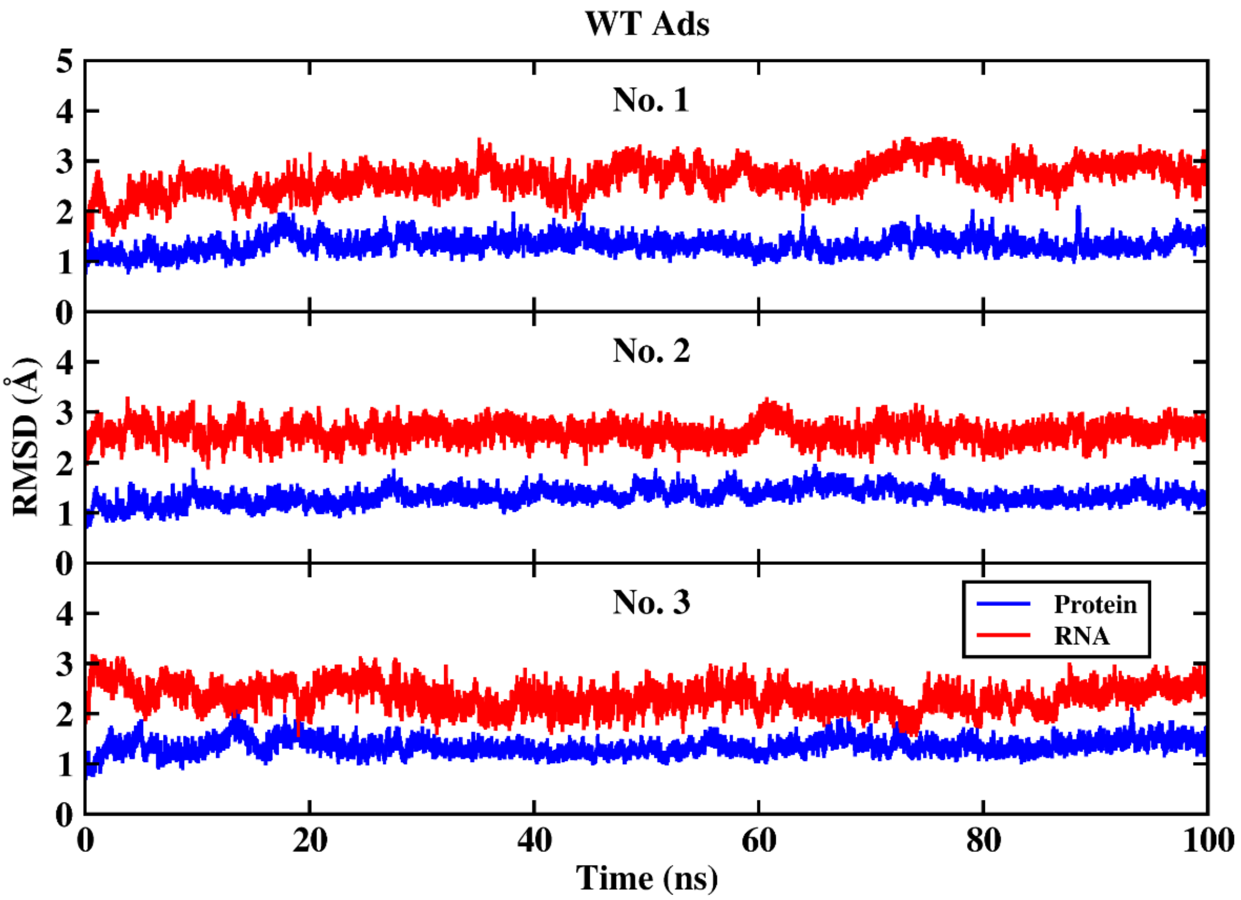

Figure S1. Protein and RNA RMSD of the Ads system from three simulations.

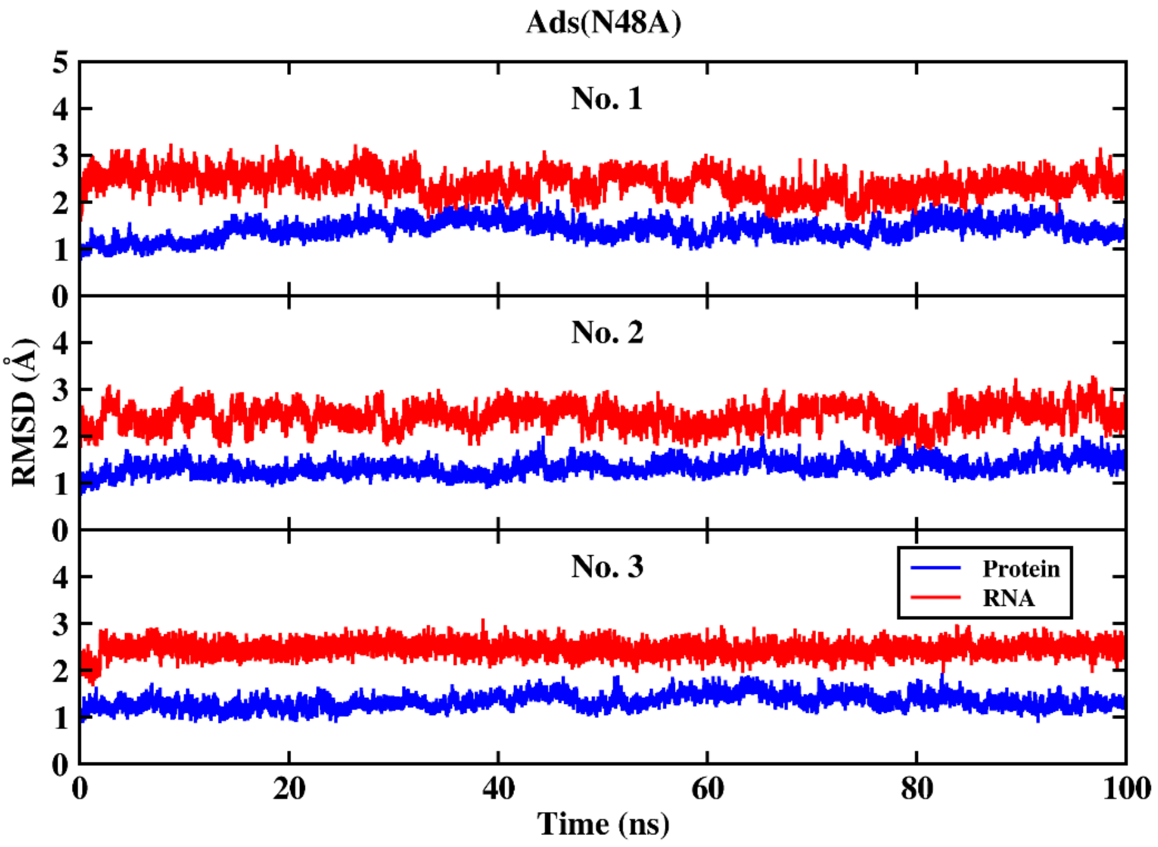

Figure S2. Protein and RNA RMSD of the Ads(N48A) system from three simulations.

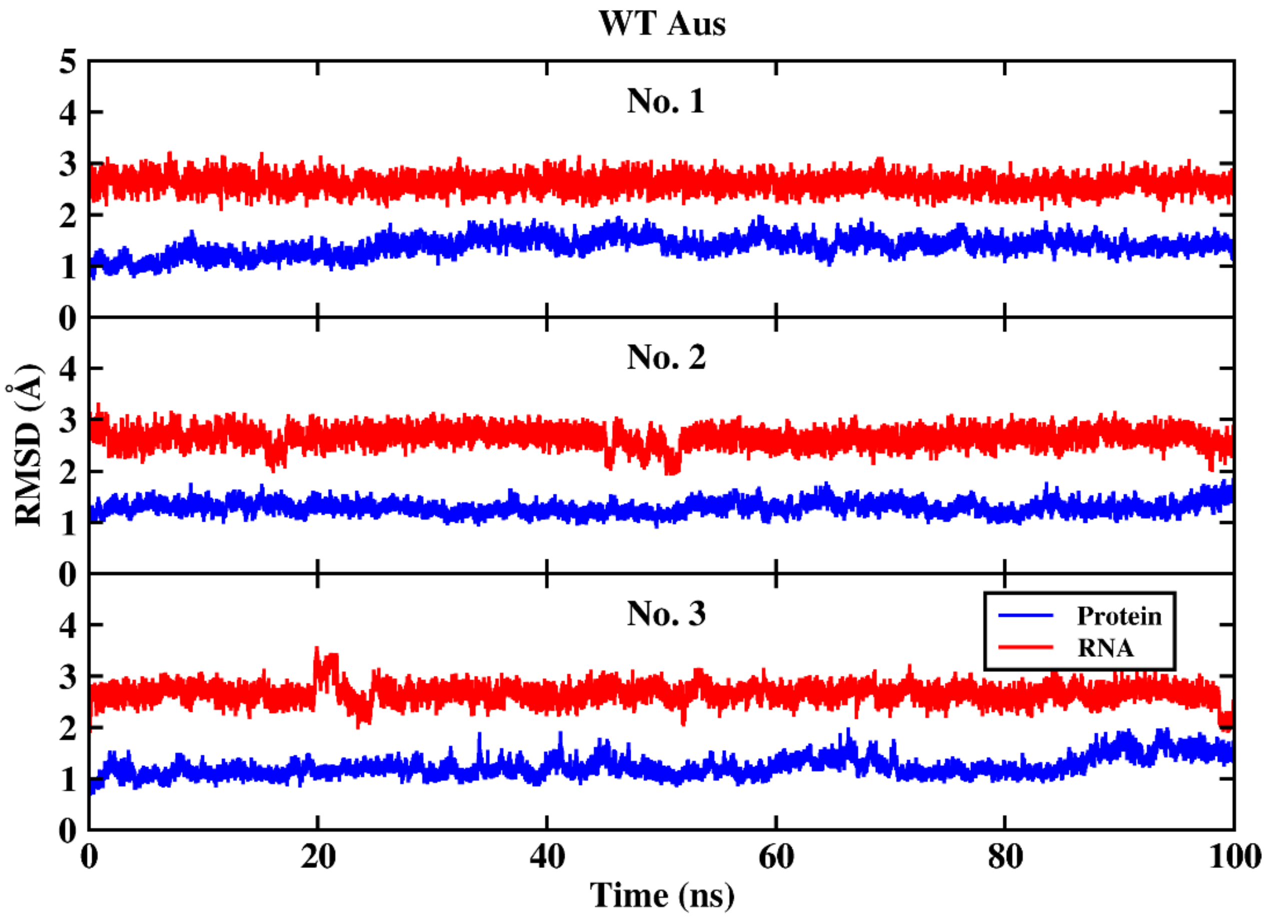

Figure S3. Protein and RNA RMSD of the Aus system from three simulations.

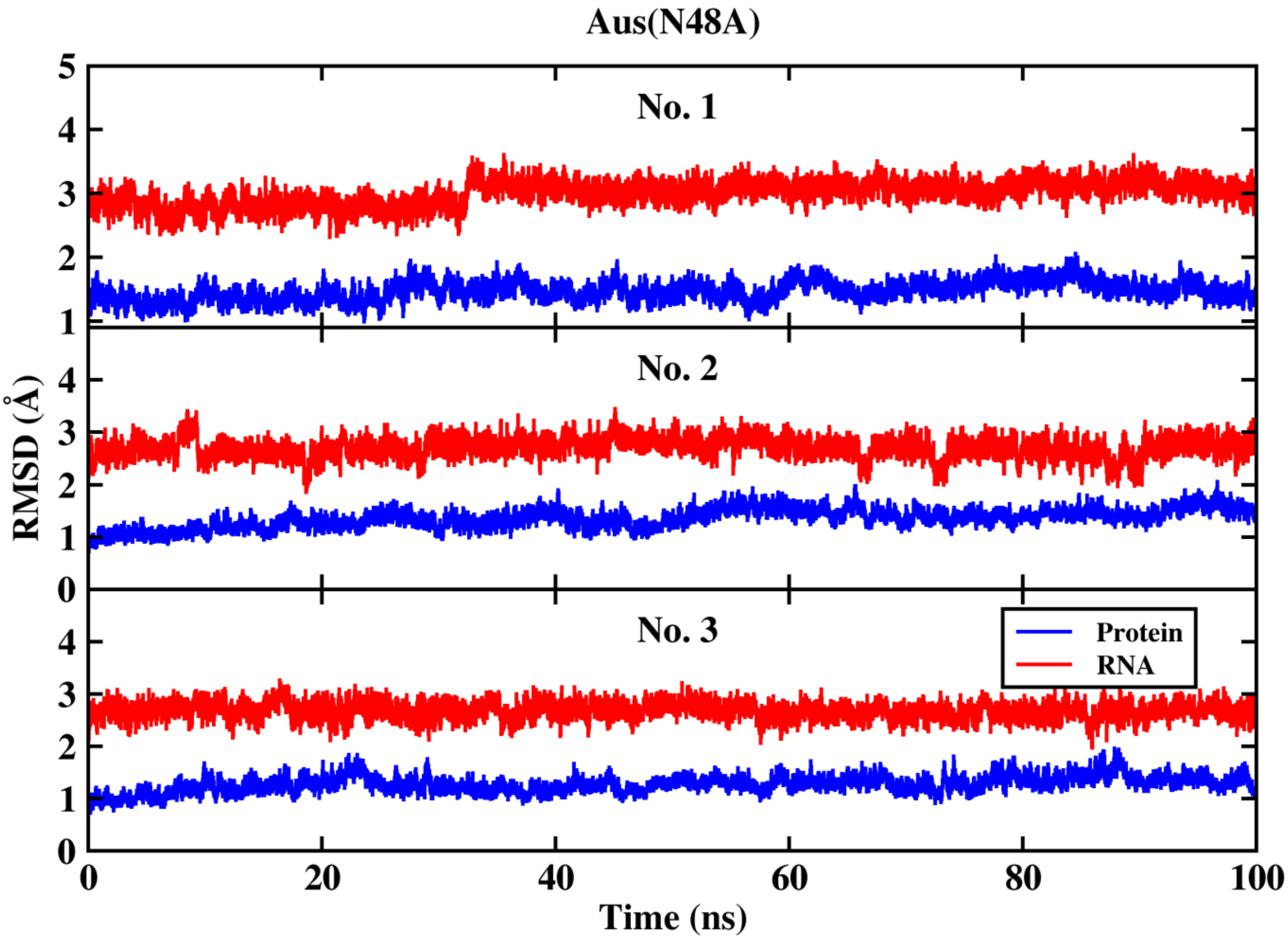

Figure S4. Protein and RNA RMSD of the Aus(N48A) system from three simulations.

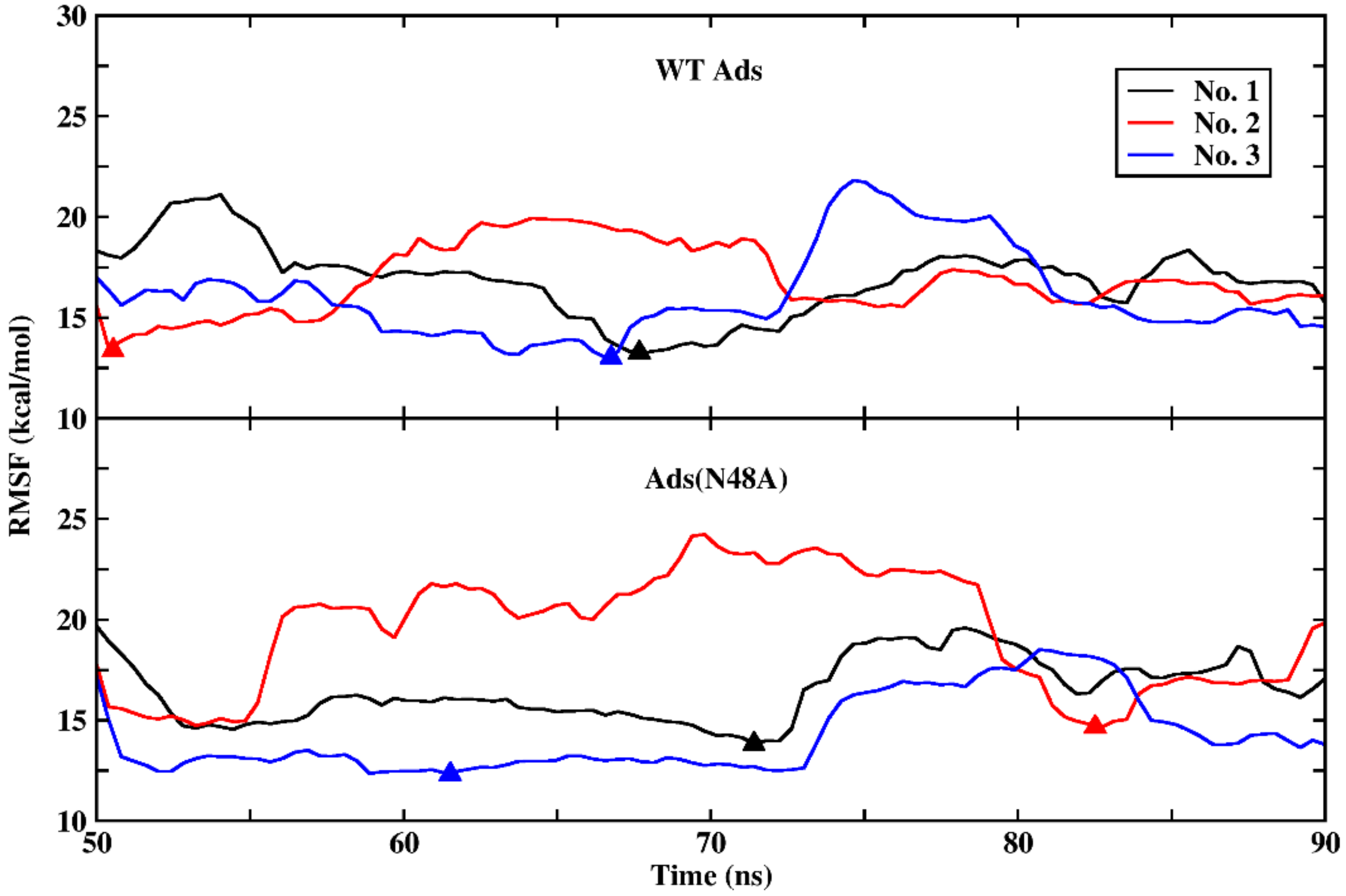

Figure S5. The RMSF of gas phase interaction energy of 10-ns sliding window in the last 50 ns for Ads and Ads(N48A) systems. The x-axis represents the start time of a 10-ns window. The windows with the smallest RMSF are marked with triangles.

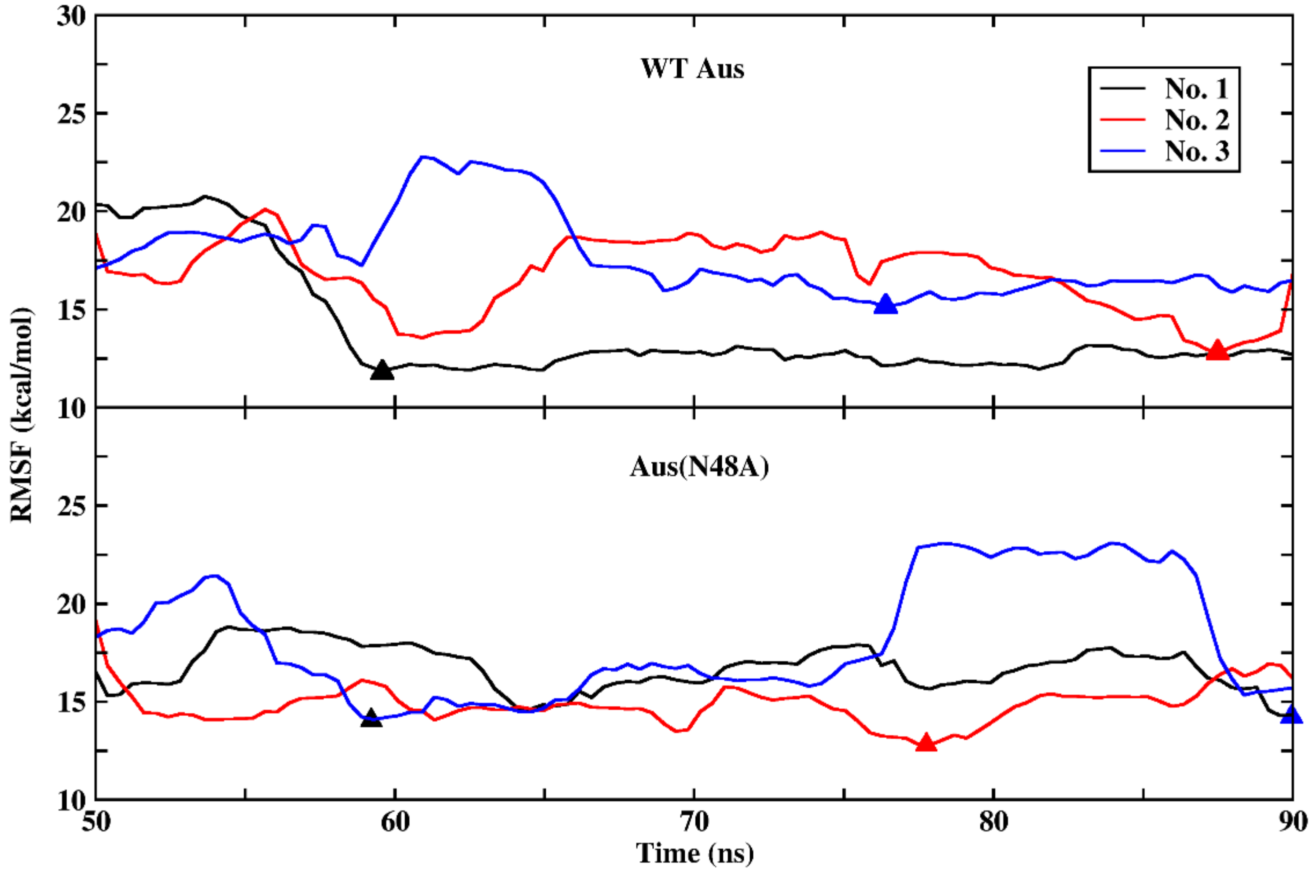

Figure S6. The RMSF of gas phase interaction energy of 10-ns sliding window in the last 50 ns for Aus and Aus(N48A) systems. The x-axis represents the start time of a 10-ns window. The windows with the smallest RMSF are marked with triangles.

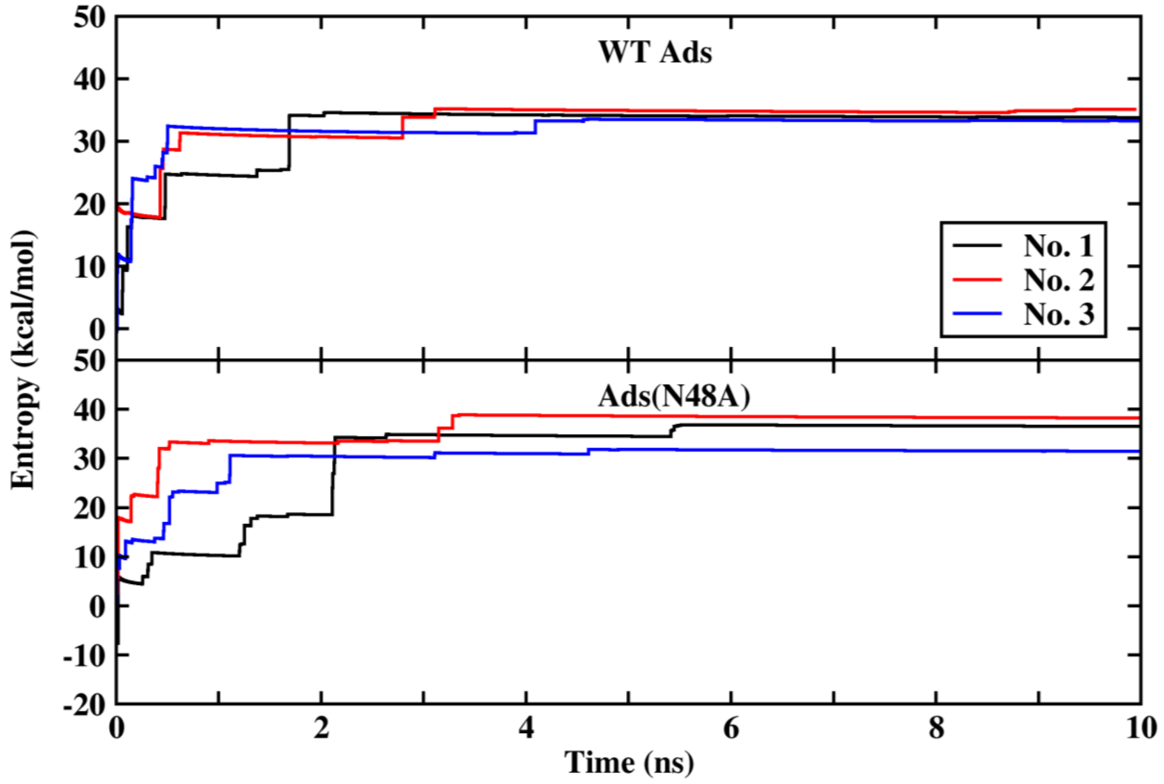

Figure S7. Time-averaged interaction entropy of continuous 10 ns with lowest energy fluctuation of for Ads and Ads(N48A) systems under three simulations.

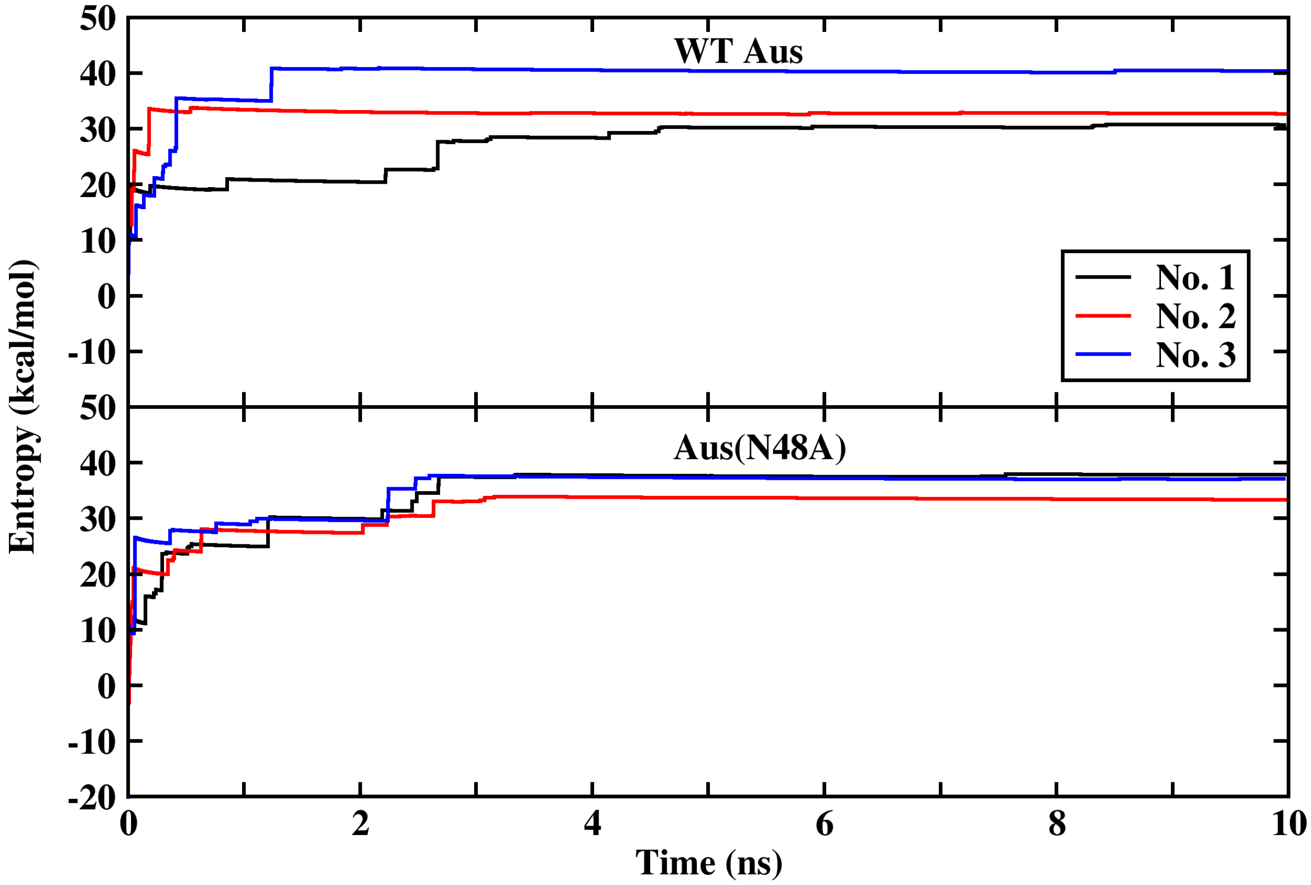

Figure S8. Time-averaged interaction entropy of continuous 10 ns with lowest energy fluctuation of for Aus and Aus(N48A) systems under three simulations.
